# Association between the Prognostic Nutritional Index and Chronic Microvascular Complications in Patients with Type 2 Diabetes Mellitus

**DOI:** 10.3390/jcm12185952

**Published:** 2023-09-13

**Authors:** Gulali Aktas

**Affiliations:** Department of Internal Medicine, Abant Izzet Baysal University Hospital, 14280 Bolu, Turkey; draliaktas@yahoo.com

**Keywords:** prognostic nutritional index, type 2 diabetes mellitus, diabetic nephropathy, diabetic neuropathy, diabetic retinopathy, inflammation

## Abstract

The prognostic nutritional index (PNI) is associated with inflammatory conditions. Since type 2 diabetes mellitus (T2DM) and its microvascular complications produce a significant inflammatory burden, we aimed to compare the PNI levels of the subjects with T2DM to those of healthy individuals. Furthermore, we aimed to compare the PNI levels of the diabetic subjects, with and without microvascular complications. The study cohort consisted of T2DM patients and healthy volunteers. The general characteristics, laboratory data, and PNI of the T2DM and control groups were compared. We further compared the PNI levels of the diabetic patients, with and without diabetic microvascular complications. The PNI levels of the T2DM patients and the control group were 51.6 (30.1–73.8)% and 64.8 (49.4–76)%, respectively (*p* < 0.001). Subgroup analyses revealed that the PNI was lower in the diabetic subjects with diabetic microvascular complications than in the diabetic patients without microvascular complications (*p* < 0.001), in patients with diabetic nephropathy compared to those without nephropathy (*p* < 0.001), in patients with diabetic retinopathy compared to those without retinopathy (*p* < 0.001), and in patients with diabetic neuropathy compared to those without neuropathy (*p* < 0.001). In conclusion, we assert that assessing the PNI may yield additional diagnostic value in regards to the timely determination of diabetic microvascular complications.

## 1. Introduction

Type 2 diabetes is increasingly prevalent worldwide. Both type 2 diabetes alone, along with its complications, place a significant burden on patients and health systems. Early diagnosis and treatment of the disease can slow down or even prevent the development of diabetes-related complications. Therefore, the early recognition of diabetic complications is essential. Chronic microvascular complications of diabetes include diabetic peripheral polyneuropathy, diabetic kidney disease, and diabetic retinopathy. The development of these complications is associated with increased morbidity and mortality in type 2 diabetes patients.

The prognostic nutritional index (PNI) is a new clinical marker with diagnostic and prognostic value in many diseases marked by inflammation. It has recently been shown that the PNI value makes a valuable contribution to predicting mortality in COVID-19 patients [1]. Type 2 diabetes is also associated with a chronic, low-grade inflammatory burden [2]. Moreover, the contribution of inflammatory parameters in diabetic patients with microvascular complications has been shown in a recent study.

Serum albumin and blood lymphocyte levels are two clinical markers used to calculate PNI. While serum albumin is a negative reactant of the acute phase of inflammation, a low lymphocyte count has been associated with poor prognosis in many diseases [3]. Therefore, it is suggested that the PNI value obtained by the combination of these two markers may be a similar and perhaps better clinical indicator of inflammation.

There is no data regarding the association between PNI and diabetic microvascular complications in type 2 diabetes patients. Hence, in the present study, we aimed to compare the PNI value in type 2 diabetes patients with and without diabetic microvascular complications.

## 2. Materials and Methods

Patients who were treated in the internal medicine outpatient clinics of our hospital and were diagnosed with type 2 diabetes were enrolled in the present retrospective study, which was granted ethical approval from local ethics committee (approval number: 2023/241, date: 18 July 2023). Diabetic patients were divided into two groups: those with and without microvascular complications. Patients who visited our outpatient clinics for routine check-ups and were found to be healthy were included in the study as a control group. Patients under the age of 18, pregnant women, those with type 1 diabetes, those who had experienced an acute infection or inflammatory disease in the last month, those with a chronic infection or inflammatory disease, and cancer patients were excluded from the study. The general characteristics of the study population (age, gender, duration of diabetes, etc.), examination findings (systolic and diastolic blood pressure, height, weight, body mass index (BMI), presence of diabetic complications, etc.), and laboratory data (blood urea, creatinine, glucose, glycosylated hemoglobin (HbA1c), estimated glomerular filtration rate (eGFR), C-reactive protein (CRP), albumin, serum lipids, aspartate and alanine transaminases (AST, ALT), and hemogram markers: leukocyte and platelet counts, hemoglobin (Hb), hematocrit (Htc), and lymphocyte count) were obtained and recorded from the institutional database and patient files. The PNI value was calculated using the formula (10 × serum albumin [g/dL]) + (0.005 × blood lymphocyte count [k/mm^3^]). The general characteristics, examination findings, and laboratory data of the diabetic subjects and controls were compared. Furthermore, data of the diabetics with microvascular complications, diabetics without microvascular complications, and controls were compared. In subgroup analysis, data of the type 2 diabetes mellitus patient were compared in terms of the presence or absence of nephropathy, retinopathy, and neuropathy.

Statistical analyses were conducted using statistical software (SPSS 16.0, IBM Co. Chicago, IL, USA). The Kolmogorov–Smirnov test was used to determine whether the study data complied with the normal distribution. Normally distributed data were expressed as means ± standard deviations and were compared between groups using the one-way ANOVA test (three-group comparison) or the independent samples t test (two-group comparison). Data that did not fit the normal distribution were expressed as medians (min-max) and were compared between groups using the Kruskal–Wallis test (for three-group comparison) or the Mann–Whitney U test (for two-group comparison). The Chi-square test was used to compare categorical variables, and these were expressed as numbers (%). The correlation between the study parameters was investigated using the Pearson correlation analysis test. The sensitivity and specificity of the level of the PNI value in determining type 2 diabetes mellitus and demonstrating microvascular diabetic complications were examined using the ROC analysis test. The binary logistic regression analysis test was used in determining whether PNI was an independent risk factor for the presence of type 2 diabetes mellitus and its microvascular complications. The statistical significance level was set at a *p* level lower than 0.05.

## 3. Results

The study population consisted of 1127 subjects: 873 patients with type 2 diabetes mellitus and 254 healthy controls. Of the diabetic patients, 426 had at least one diabetic microvascular complication, while 447 were free from these complications.

### 3.1. Analyses of the Data of Diabetic and Control Subjects

The mean ages of the diabetics and control individuals in the study were 57.7 ± 10.6 years and 47.2 ± 13.8 years, respectively (*p* < 0.001). A total of 416 (47.7%) diabetic patients were women, and 457 (52.3%) of them were men, while 73 (28.7%) of the control subjects were women, and 181 (71.3%) of them were men. Gender was statistically different between type 2 diabetes mellitus patients and the healthy controls (*p* < 0.001). The median PNI levels of the diabetic patients and the control group were 51.6 (30.1–73.8)% and 64.8 (49.4–76)%, respectively. The PNI of the patients with type 2 diabetes mellitus was significantly decreased compared to the PNI of the control subjects (*p* < 0.001). The mean Hb and Htc values and the median height, systolic and diastolic blood pressures, leukocyte and platelet counts, serum creatinine, and the AST and ALT levels of the diabetes mellitus and control groups were not statistically different (*p* > 0.05 for all). However, the median weight, waist circumference, BMI, serum albumin, C-reactive protein, HbA1c, fasting glucose, blood urea, eGFR, and serum lipids of the type 2 diabetes mellitus group were significantly different from those of the control subjects (*p* < 0.05 for all). The rates of the subjects who smoked (*p* < 0.001) and drank alcohol (*p* < 0.001) were both higher in the diabetes mellitus group compared to the control group. Table 1 summarizes the data of the type 2 diabetes mellitus and control groups.

Correlation tests revealed that PNI was significantly and inversely correlated with age (r = −0.25, *p* < 0.001), waist circumference (r = −0.39, *p* < 0.001), body mass index (r = −0.11, *p* < 0.001), C-reactive protein (r = −0.26, *p* < 0.001), HbA1c (r = −0.4 *p* < 0.001), and fasting blood glucose (r = −0.38, *p* < 0.001). In addition, PNI was positively correlated with eGFR level (r = 0.24, *p* < 0.001).

**Figure 1:**
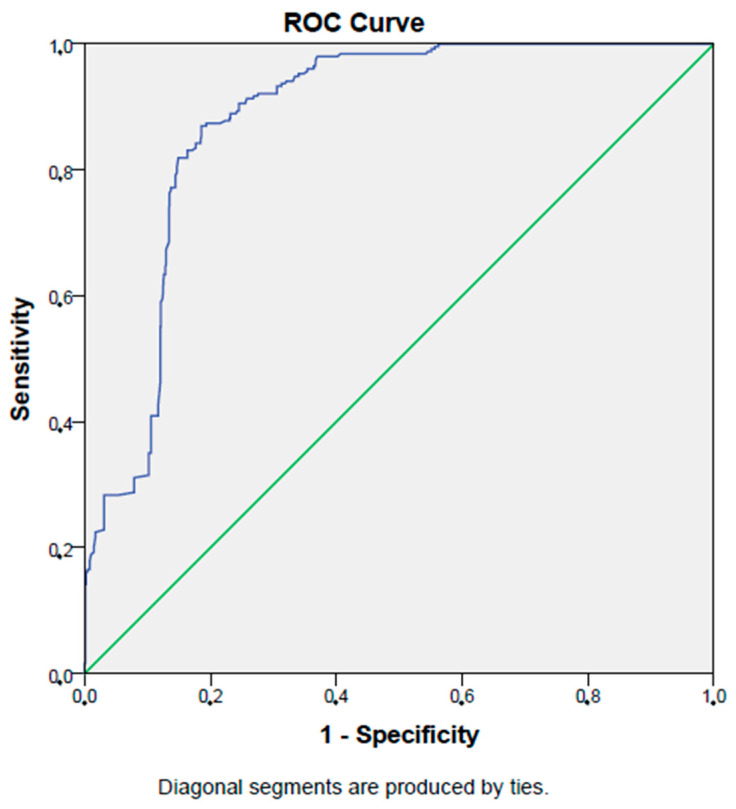
ROC curve of PNI in detecting type 2 DM.

In ROC analysis, a PNI level lower than 60.5% had an 87% sensitivity and an 81% specificity in selecting patients with type 2 diabetes mellitus (AUC:0.88, *p* < 0.001, 95% CI: 0.86–0.90). Figure 1 shows the ROC curve of the PNI in detecting type 2 diabetes mellitus patients.

A logistic regression analysis model considering age, gender, body mass index, waist circumference, serum creatinine, eGFR, CRP, HbA1c, and fasting blood glucose, along with PNI, showed that PNI was an independent risk factor for type 2 diabetes mellitus, since a unit decrease in PNI increased the odds of type 2 diabetes by 1.46 times (*p* < 0.001, OR: 1.46, 95% CI: 1.27–1.68), (Table 2).

### 3.2. Analyses of the Data of Diabetic Subjects with/without Microvascular Complications and Control Subjects

Comparison of the data of the diabetic patients with microvascular complications (n = 426), those without microvascular complications (n = 447), and the control subjects (n = 254) revealed that the mean age of the diabetic patients with microvascular complications (59.3 ± 10.5 years) was significantly higher than the age of the diabetic patients without microvascular complications (56.2 ± 10.4 years) and the control subjects (47.2 ± 13.8 years), (*p* < 0.001). There were 253 (59%) women and 173 (41%) men in the diabetic microvascular complications group, 163 (36.5%) women and 284 (63.5%) men in the diabetic patients with microvascular complications group, and 73 (28.7%) women and 181 (71.3%) men in the control group (*p* < 0.001). The median PNI levels of the diabetic patients with microvascular complications, diabetic subjects without microvascular complications, and controls were 42.8 (30.1–62.5)%, 57.6 (43.1–73.8), and 64.8 (49.4–76)%, respectively (*p* < 0.001). The PNI of the controls was significantly higher than that of the diabetic patients, either with or without microvascular complications. The median height, systolic and diastolic blood pressures, leukocyte and platelet counts, blood urea, creatinine, and AST and ALT levels of the diabetes mellitus patients with microvascular complications were not statistically different than those of the diabetic patients without microvascular complications and those of the control group (*p* > 0.05 for all). However, the mean blood Hb, median weight, waist circumference, BMI, blood Htc, serum albumin, C-reactive protein, HbA1c, fasting glucose, eGFR, and serum lipids of these three groups were significantly different (*p* < 0.05 for all). Table 3 summarizes the data of the diabetes mellitus patients with microvascular complications, diabetic patients without microvascular complications, and control group.

**Figure 2:**
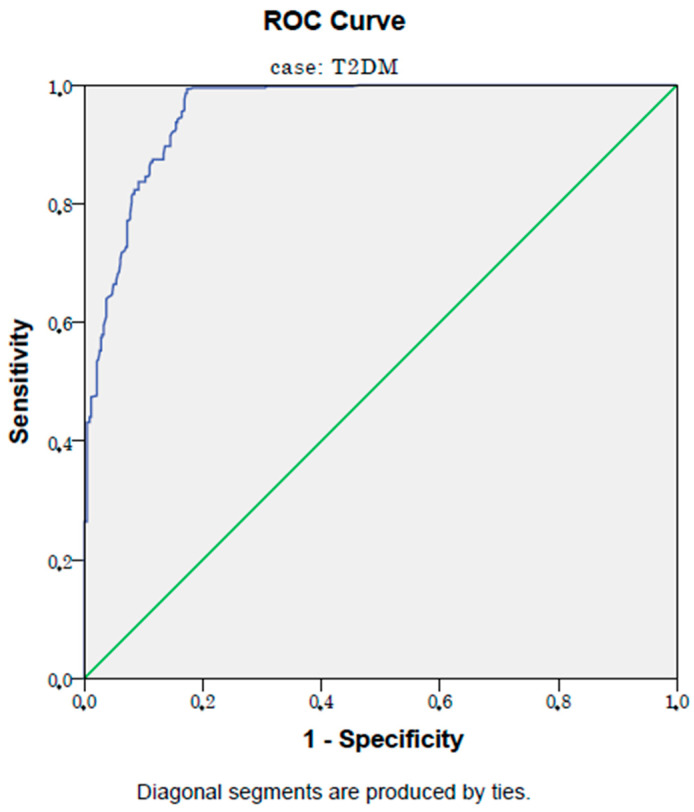
ROC curve of PNI in detecting microvascular complications.

In ROC analysis, a PNI level lower than 48.5% has a 99% sensitivity and an 83% specificity in detecting type 2 diabetes mellitus patients with microvascular complications (AUC:0.96, *p* < 0.001, 95% CI: 0.94–0.97). Figure 2 shows the ROC curve of the PNI in detecting type 2 diabetes mellitus patients with microvascular complications.

A logistic regression analysis model considering age, gender, body mass index, waist circumference, serum creatinine, eGFR, CRP, HbA1c, and fasting blood glucose, along with PNI, showed that PNI was an independent risk factor for microvascular complications in type 2 diabetes mellitus, since a unit decrease in PNI increased the odds of diabetic microvascular complication by 1.58 times (*p* < 0.001, OR: 1.58, 95% CI: 1.47–1.70).

### 3.3. Analyses of the Data of Diabetic Subjects with/without Diabetic Nephropathy

General characteristics and laboratory data of the type 2 diabetes mellitus patients with diabetic nephropathy (n = 307) and those without diabetic nephropathy (n = 566) were compared, and the median ages of the diabetic subjects with and without diabetic nephropathy were found to be 57 (36–87) years and 60 (29–89) years, respectively (*p* = 0.42). A total of 164 (53%) of the subjects were women, and 143 (47%) were men in the diabetic patients with diabetic nephropathy group, while 252 (44.5%) of the subjects were women, and 314 (55.5%) were men in the diabetic patients without diabetic nephropathy group (*p* = 0.01). The median PNI levels of the diabetic patients, with and without diabetic nephropathy, were 43 (30–63)%, and 56 (36–74)%, respectively (*p* < 0.001). The median height, systolic and diastolic blood pressures, blood leukocyte count, blood urea, creatinine, and AST and ALT levels of the diabetes mellitus patients with diabetic nephropathy were not statistically different than those of the subjects without diabetic nephropathy (*p* > 0.05 for all). On the other hand, the median weight, waist circumference, BMI, blood Hb and Htc, blood platelet count, serum albumin, C-reactive protein, HbA1c, fasting glucose, eGFR, and serum lipids of the patients, with and without diabetic nephropathy, were significantly different (*p* < 0.05 for all). Table 4 shows the data of the diabetic patients, with and without diabetic nephropathy.

**Figure 3:**
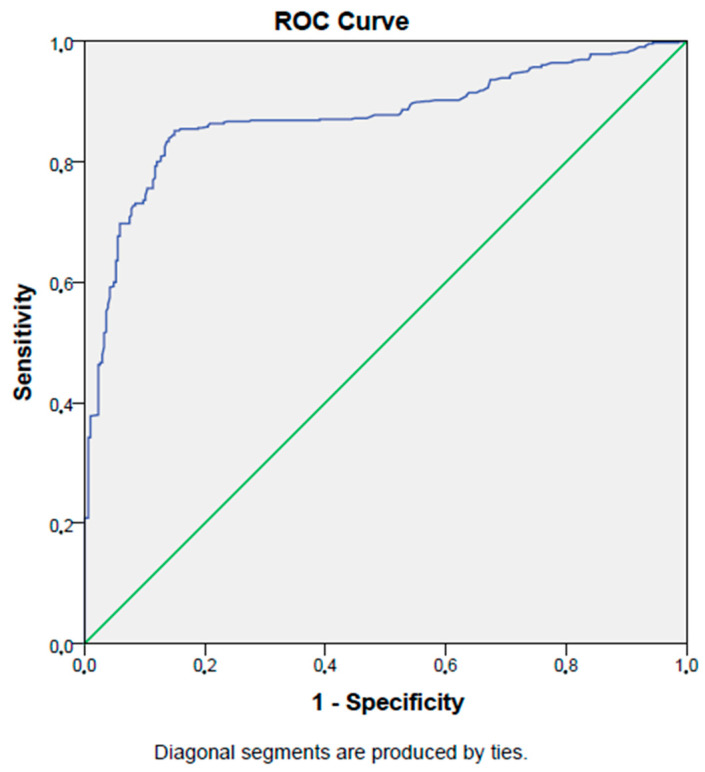
ROC curve of PNI in detecting diabetic nephropathy.

In ROC analysis, a PNI level lower than 48.3% has an 84% sensitivity and an 86% specificity in detecting diabetic nephropathy in type 2 diabetes mellitus patients (AUC:0.87, *p* < 0.001, 95% CI: 0.85–0.90). Figure 3 shows the ROC curve of the PNI in detecting diabetic nephropathy in type 2 diabetes mellitus patients.

A logistic regression analysis model considering age, gender, body mass index, waist circumference, serum creatinine, eGFR, CRP, HbA1c, and fasting blood glucose, along with PNI, showed that PNI was an independent risk factor for diabetic nephropathy in type 2 diabetes mellitus, since a unit decrease in PNI increased the odds of diabetic nephropathy by 1.29 times (*p* < 0.001, OR: 1.29, 95% CI: 1.25–1.34).

### 3.4. Analyses of the Data of Diabetic Subjects with/without Diabetic Retinopathy

The general characteristics and laboratory data of the type 2 diabetes mellitus patients with diabetic retinopathy (n = 81) and those without diabetic retinopathy (n = 792) were compared, and the median ages of the diabetic subjects, with and without diabetic retinopathy, were found to be 56 (46–89) years, and 60 (29–86) years, respectively (*p* = 0.66). A total of 60 (74%) of the subjects were women, and 21 (26%) were men in the diabetic patients with diabetic retinopathy group, while 356 (45%) of the subjects were women, and 436 (55%) were men in the diabetic patients without diabetic retinopathy group (*p* < 0.001). The median PNI levels of the diabetic patients, with and without diabetic retinopathy, were 43.3 (36.3–59.8)%, and 52.7 (30.1–73.8)%, respectively (*p* < 0.001). The median weight, waist circumference, BMI, systolic and diastolic blood pressures, blood leukocyte and platelet counts, blood urea, creatinine, eGFR, AST, ALT, and serum lipid levels of the diabetes mellitus patients with diabetic retinopathy were not statistically different from those of the subjects without diabetic retinopathy (*p* > 0.05 for all). On the other hand, the median height, blood Hb and Htc, serum albumin, C-reactive protein, HbA1c, and fasting glucose of the patients with and without diabetic retinopathy were significantly different (*p* < 0.05 for all). Table 5 shows the data of the diabetic patients, with and without diabetic retinopathy.

**Figure 4:**
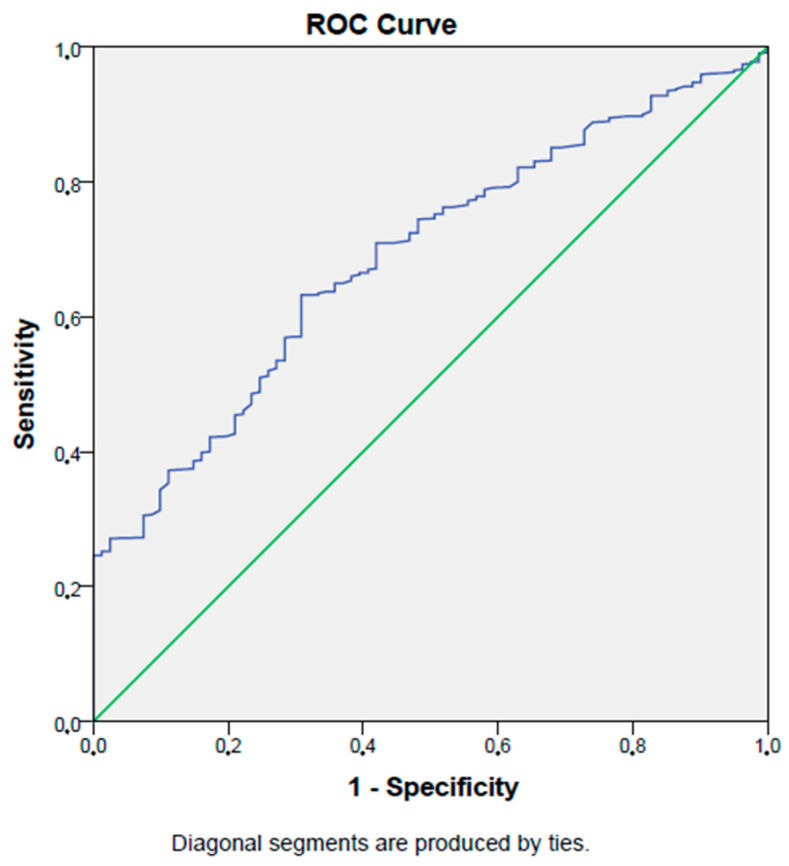
ROC curve of PNI in detecting diabetic retinopathy.

In the ROC analysis, a PNI level lower than 47.7% has a 63% sensitivity and a 69% specificity in detecting diabetic retinopathy in type 2 diabetes mellitus patients (AUC: 0.69, *p* < 0.001, 95% CI: 0.64–0.74). Figure 4 shows the ROC curve of the PNI in detecting diabetic retinopathy in type 2 diabetes mellitus patients.

A logistic regression analysis model considering age, gender, body mass index, waist circumference, serum creatinine, eGFR, CRP, HbA1c, and fasting blood glucose, along with PNI, showed that PNI was an independent risk factor for diabetic retinopathy in type 2 diabetes mellitus patients, since a unit decrease in PNI increased the odds of diabetic retinopathy by 106% (*p* = 0.001, OR: 1.068, 95% CI: 1.03–1.10).

### 3.5. Analyses of the Data of Diabetic Subjects with/without Diabetic Neuropathy

There were 288 subjects in the diabetic neuropathy group, while there were 585 diabetic patients without neuropathy. The median ages of the diabetic subjects, with and without diabetic neuropathy, were 58.5 (41–87) years and 60 (29–89) years, respectively (*p* = 0.02). A total of 184 (64%) subjects were women, and 104 (36%) were men in the diabetic patients with diabetic neuropathy group, while 232 (40%) of the subjects were women, and 353 (60%) were men in the diabetic patients without diabetic neuropathy group (*p* < 0.001). The median PNI levels of the diabetic patients, with and without diabetic neuropathy, were 42.4 (30.1–62.5)% and 55.3 (36.3–73.8)%, respectively (*p* < 0.001). The median waist circumference, BMI, systolic and diastolic blood pressures, blood leukocyte and platelet counts, blood urea, creatinine, serum triglyceride, and AST and ALT levels of the diabetes mellitus patients with diabetic neuropathy were not statistically different than those of the subjects without diabetic neuropathy (*p* > 0.05 for all). On the other hand, the median height, weight, blood Hb and Htc, eGFR, serum albumin, C-reactive protein, HbA1c, fasting glucose, serum total, and LDL and HDL cholesterol levels of the patients, with and without diabetic neuropathy, were significantly different (*p* < 0.05 for all). Table 6 shows the data for the diabetic patients, with and without diabetic neuropathy.

**Figure 5:**
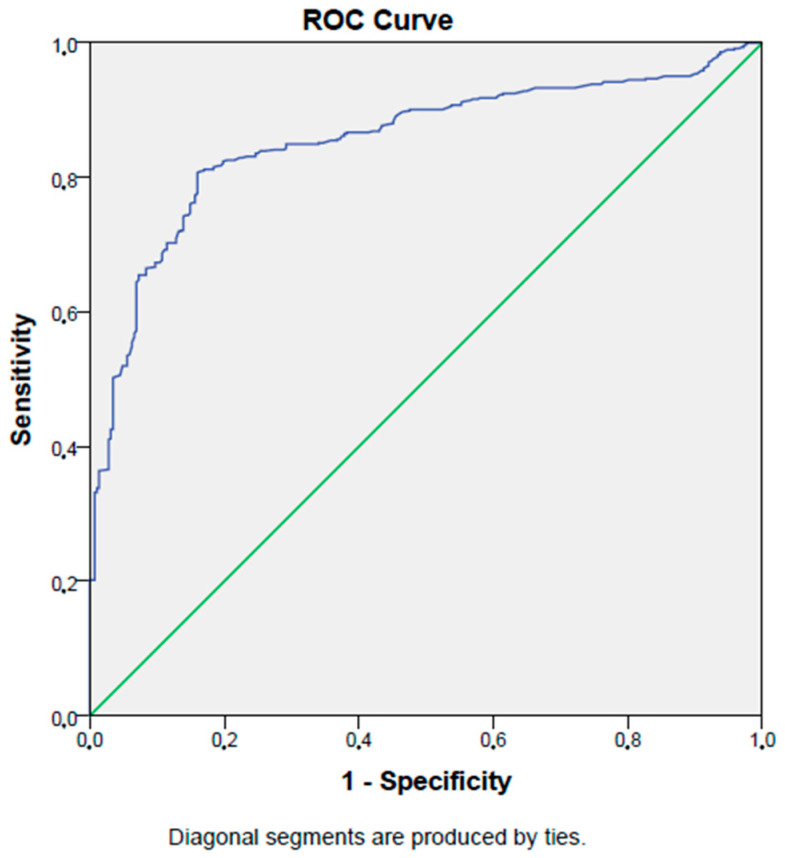
ROC curve of PNI in detecting diabetic neuropathy.

In ROC analysis, a PNI level lower than 48.5% has an 81% sensitivity and an 84% specificity in detecting diabetic neuropathy in type 2 diabetes mellitus patients (AUC:0.85, *p* < 0.001, 95% CI: 0.834–0.88). Figure 5 shows the ROC curve of the PNI in detecting diabetic neuropathy in type 2 diabetes mellitus patients.

A logistic regression analysis model considering age, gender, body mass index, waist circumference, serum creatinine, eGFR, CRP, HbA1c, and fasting blood glucose, along with PNI, showed that PNI was an independent risk factor for diabetic neuropathy in type 2 diabetes mellitus patients, since a unit decrease in PNI increased the odds of diabetic neuropathy by 1.23 times (*p* < 0.001, OR: 1.23, 95% CI: 1.19–1.27).

## 4. Discussion

The remarkable results of the present work could be summarized as follows: (a) the PNI of the type 2 diabetes mellitus patients was significantly lower than the PNI of the healthy controls; (b) PNI was inversely correlated with body mass index, HbA1c, and fasting blood glucose, while it was positively correlated with eGFR; (c) PNI has a considerably high detection capacity for type 2 diabetes mellitus, with an 87% sensitivity and an 81% specificity; (d) PNI was an independent risk factor for type 2 diabetes mellitus, since a unit decrease in PNI increased the odds of type 2 diabetes by 146%; (e) the PNI of the diabetic subjects with microvascular complications was significantly lower than the PNI of both diabetic patients without microvascular complications and the PNI of the healthy controls; (f) decreased PNI exhibits a significant sensitivity and specificity (99% and 83%, respectively) in detecting type 2 diabetes mellitus patients with microvascular complications; (g) PNI was an independent risk factor for microvascular complications in type 2 diabetes patients, since a unit decrease in PNI increased the odds of diabetic microvascular complications by 158%; (h) the PNI of the diabetic patients with nephropathy was significantly reduced compared to that of the diabetic subjects without nephropathy; (i) reduced PNI has an 84% sensitivity and an 86% specificity in selecting diabetic patients with nephropathy; (j) decreased PNI was an independent risk factor for diabetic nephropathy, since a unit reduction in PNI increased the odds of diabetic nephropathy by 129%; (k) the PNI of the diabetic patients with retinopathy was significantly reduced compared to that of the diabetic subjects without retinopathy; (l) low PNI has a moderate sensitivity and specificity (63% and 69%, respectively) in detecting diabetic retinopathy; (m) a reduction in PNI was an independent risk factor for diabetic retinopathy because a unit decrease in PNI increased the odds of diabetic retinopathy by 106% in patients with type 2 diabetes mellitus; (n) the PNI of the diabetic patients with neuropathy was significantly lower than the PNI of the diabetic subjects without neuropathy; (o) low PNI exhibits considerable sensitivity and specificity (81% and 84%, respectively) in selecting diabetic patients with neuropathy; and (p) a reduction in PNI was an independent risk factor for diabetic neuropathy because a unit decrease in PNI increased the odds of neuropathy by 123%.

Recent works on the pathogenesis of type 2 diabetes mellitus revealed a significant association between inflammation and the disease. Elevated blood glucose, oxidative stress driven by hyperglycemia, inflammation, and development of type 2 diabetes were linked to each other, as suggested by a recent review [4]. Besides being the most common and debilitating metabolic disorder, type 2 diabetes mellitus is also associated with chronic, low-grade inflammatory burden [5]. For example, Keeter et al. emphasized the role of neutrophils, the most common leukocyte type in the bloodstream, in type 2 diabetes mellitus and in its atherosclerotic complications [6]. Insulin resistance in type 2 diabetes patients is responsible of the production and release of several cytokines, leading to the hepatic overproduction of acute phase proteins such as C- reactive protein, serum amyloid A, and plasminogen activator inhibitor-1 [7]. These inflammatory markers have been linked with diabetes, as well as the early phase of the disease, known as prediabetes [8]. On the other hand, PNI has also been linked with inflammatory diseases. It has been proposed as a prognostic marker in various types of cancer, including lung cancer [9], renal cell cancer [10], colorectal cancer [11], hepatoma [12], and gastric cancer [13]. Additionally, decreased PNI has been suggested as a marker of disease severity in COVID-19 infection [14]. Moreover, Wada et al. reported that PNI was a useful marker for long term clinical outcomes in patients with stable coronary artery disease [15]. All of these conditions—cancer, cardiovascular diseases, and infections—are characterized by some degree of inflammation, as is type 2 diabetes. Accordingly, we reported decreased levels of PNI in type 2 diabetes mellitus patients compared to those of the healthy controls, which is consistent with the literature data, since PNI is considered as a novel inflammatory marker.

The present study showed that microvascular complications of type 2 diabetes were associated with lower PNI values. Furthermore, PNI was very sensitive and specific in the detection of microvascular diabetic complications. This is not surprising when we consider that PNI is reduced in diseases characterized by inflammation, and that microvascular diabetic complications are also associated with an increased burden of inflammation. Various inflammatory pathways are involved in the pathogenesis of diabetic microvascular complications. For example, non-enzymatic glycation and the formation of advanced glycation end products, oxidative stress, polyol and diacylglycerol-protein kinase C pathways, and immune dysregulation/inflammation play important roles in the development of diabetic complications [16]. Similarly, metabolic markers of the inflammatory burden have been reported to be higher in patients with diabetic microvascular complications than in diabetic subjects without microvascular complications [17]. These literature data are in agreement with the finding of lower PNI levels in patients with diabetic microvascular complications compared to those without complications and to healthy controls found in the present work.

Diabetic nephropathy, specifically diabetic kidney disease or diabetic kidney injury, is one of the most common causes of deterioration in kidney function, and is the leading cause of end stage kidney failure [18]. Diabetic nephropathy is associated with greater inflammation than that found in type 2 diabetes mellitus itself. Higher levels of serum uric acid have been reported in diabetic patients with nephropathy compared to those without nephropathy [17]. A more recent study revealed that the uric acid/HDL cholesterol ratio, a novel inflammatory marker, was increased in patients with diabetic kidney injury compared to the diabetic patients without diabetic kidney injury [19]. These novel inflammatory markers are considered as emerging biomarkers of diabetic kidney disease, making them promising therapeutic targets. Not only novel inflammatory biomarkers, but also well-established predictors of inflammation, are increased in diabetic subjects with nephropathy. Bilgin et al. compared laboratory markers of diabetic nephropathy patients to those of diabetes mellitus patients without nephropathy and reported significantly increased C-reactive protein levels in diabetic subjects with nephropathy compared to type 2 diabetes mellitus patients without diabetic nephropathy [20]. Moreover, serum amyloid A, another commonly assessed marker of acute phase reaction, has been reported to be a risk factor of mortality in patients with diabetic kidney disease [21]. In another study, besides CRP, TNF was also found to be an independent predictor of diabetic kidney disease in type 2 diabetes patients [22]. These data suggest that diabetic nephropathy is associated with a significant burden of inflammation. In accordance with the literature, we found decreased PNI levels in diabetic nephropathy subjects compared to those of diabetic patients without nephropathy.

In the present study, decreased PNI levels were found in diabetic neuropathy subjects compared to diabetic patients without neuropathy. There are similar results in the literature. Diabetic neuropathy is characterized by low-grade chronic inflammation at the subclinical level [23]. Besides inflammatory pathways, oxidative stress is also involved in the pathogenesis of diabetic neuropathy [24]. The serum C-reactive protein level has been linked with impairments related to diabetic neuropathy in diabetic subjects [25]. Moreover, increased C-reactive protein levels were noted in subjects with diabetic neuropathy when compared to those without diabetic neuropathy [26]. Subsequently, a recent study confirmed the previous works by reporting increased C-reactive protein levels in diabetic neuropathy subjects compared to those of the diabetic patients without neuropathy [27]. Another inflammatory marker, high sensitivity C-reactive protein levels, were higher in the diabetic patients with diabetic foot ulcers, a complication closely associated with diabetic neuropathy, than those in diabetic patients without foot ulcers [28]. Moreover, the association between diabetic neuropathy and tumor necrosis factor-alpha has been suggested to be stronger than the association between C-reactive protein and diabetic neuropathy [29]. In an experimental animal study, it has been reported that no abnormal nerve functions were noted in tumor necrosis factor-alpha deficient diabetic rats compared to the non-diabetic rats [30]. These data confirm the association between diabetic neuropathy and the increased burden of inflammation. Since PNI is also a marker related to inflammatory conditions, its association with diabetic neuropathy, as noted in the present work, is considered an inevitable result.

Diabetic retinopathy is characterized by an inflammatory attack on the retinal vascular endothelium. It is also associated with increased serum levels of inflammatory markers. Elevated levels of serum C-reactive protein have been linked with serious diabetic retinopathy [31]. Similarly, tumor necrosis factor-alpha was suggested as an independent risk factor for diabetic retinopathy [32]. In addition, the development of diabetic retinopathy was reported to be triggered by increased serum plasminogen activator inhibitor-1 (PAI-1) and soluble endothelial leukocyte adhesion molecule-1 (sE-selectin) levels [33]. A decrease in PNI may reflect the inflammatory burden in conditions characterized by inflammation. For example, decreased PNI was reported as a poor prognostic marker in ulcerative colitis in the study of Chohno et al. [34]. Their study was confirmed by a recent work, which suggested PNI as a predictive marker for complications in patients with ulcerative colitis [35]. Hence, our results revealing lower PNI levels in patients with diabetic retinopathy than in the diabetic subjects without retinopathy are consistent with the literature data.

Here, we can discuss why PNI is a useful inflammatory marker in type 2 diabetes mellitus and diabetic microvascular complications. PNI is an index which includes serum albumin and blood lymphocyte count. Serum albumin is a negative acute phase marker that decreases during acute inflammation and infection. Moreover, it has been suggested as an independent risk factor for the progression of diabetic nephropathy [36]. Similarly, significantly decreased levels of serum albumin were reported in diabetic nephropathy subjects compared to the diabetic patients without diabetic nephropathy [20]. On the other hand, inflammatory conditions are characterized by a decrease in blood lymphocyte count. Some examples of decrease in lymphocyte count in inflammatory diseases include rheumatoid arthritis [37], COVID-19 infection [38], and Behçet’s disease [39]. However, there are also conflicting reports in the literature in regards to this topic. A recent work, which studied laboratory values of the diabetic patients with and without nephropathy, revealed slightly higher (but insignificant) blood lymphocyte counts in diabetic nephropathy patients compared to those in the subjects without nephropathy [40]. Nevertheless, low serum albumin and low blood lymphocyte levels seem to be associated with the inflammatory burden in certain conditions. PNI, a marker obtained by combining these two levels, seems to be associated with both diabetes and its microvascular complications, and is even considered as an independent risk factor for the presence of diabetic microvascular complications. PNI consists of the blood lymphocyte count and serum albumin, which are readily available in almost all medical institution laboratories. This makes PNI an easily assessable marker. Studying serum albumin and lymphocyte count is cost-effective, making PNI an inexpensive marker.

Some limitations of the present work include the retrospective and single center design of the study. However, to the best of our knowledge, this is the first study in literature to report a significant association between decreased PNI and type 2 diabetes mellitus and its microvascular complications in a considerably large cohort. The clinical significance of the results of the present study is that PNI, as an easily accessible and inexpensive tool, can be a useful in the detection of T2DM and its microvascular complications.

## 5. Conclusions

In summary, we assert that the easy to assess and inexpensive nature of PNI, combined with its significant association with type 2 diabetes and diabetic microvascular complications, make it a useful additional diagnostic tool for the detection of these conditions.

## Figures and Tables

**Table 1:** Data for the type 2 diabetes mellitus and control groups.

		T2DM Group	Control Group	*p*
Sex	Women (n, %)	416 (48%)	73 (29%)	<0.001
	Men (n, %)	457 (52%)	181 (71%)	
Smoking	Yes (n, %)	187 (21%)	28 (11%)	<0.001
	No (n, %)	688 (79%)	226 (89%)	
Alcohol consumption	Yes (n, %)	56 (6%)	0 (0%)	<0.001
	No (n, %)	817 (94%)	254 (100%)	
		*Mean ± SD*		
Age (years)		57.7 ± 10.6	47.2 ± 13.8	<0.001
Hb (g/dL)		13.8 ± 1.9	13.9 ± 1.4	0.39
Htc (%)		40.5 ± 5.4	41.5 ± 4.1	0.08
		*Median (min–max)*		
PNI (%)		51.6 (30.1–73.8)	64.8 (49.4–76)	<0.001
Height (m)		1.64 (1.35–1.9)	1.67 (1.52–1.87)	0.1
Weight (kg)		86 (45–150)	72 (55–136)	<0.001
Waist circumference (cm)		105 (75–160)	95 (65–144)	<0.001
BMI (kg/m^2^)		31.1 (16.6–55.4)	27.6 (18.3–49.4)	<0.001
Systolic blood pressure (mmHg)		120 (90–200)	120 (100–180)	0.12
Diastolic blood pressure (mmHg)		75 (50–110)	80 (50–105)	0.11
Leukocyte count (k/mm^3^)		7.2 (4.2–14.4)	5.5 (4–14.1)	0.1
Platelet count (k/mm^3^)		230 (154–441)	239 (151–374)	0.64
Albumin (g/dL)		4 (1.8–5.6)	4.4 (3.9–5.4)	<0.001
C-reactive protein (mg/L)		4.1 (0.1–25)	2.4 (0.1–12)	<0.001
HbA1c (%)		7.6 (5.9–17.2)	5.4 (4.8–6.4)	<0.001
Glucose (mg/dL)		142 (65–565)	93 (69–117)	<0.001
Urea (mg/dL)		32 (13–258)	26 (13–62)	<0.001
Creatinine (mg/dL)		0.8 (0.39–3.93)	0.69 (0.4–1.3)	0.09
eGFR (%)		102 (14–111)	114 (58–118)	<0.001
AST (U/L)		19 (6–97)	18 (9–35)	0.15
ALT (U/L)		23 (6–96)	19 (6–58)	0.11
Total cholesterol (mg/dL)		204 (50–378)	194 (114–290)	<0.001
LDL-cholesterol (mg/dL)		125 (21–244)	112 (49–192)	<0.001
HDL-cholesterol (mg/dL)		44 (13–92)	48 (21–85)	<0.001
Triglyceride (mg/dL)		156 (47–1050)	124 (52–680)	<0.001

**Table 2:** Risk factors of T2DM in regression analysis.

	*p*	OR	95% CI
Age	0.53	0.98	0.93–1.04
Gender	0.48	1.7	0.39–7.36
PNI	<0.001	1.46	1.27–1.68
Waist circumference	0.87	0.99	0.91–1.08
BMI	0.01	0.72	0.55–0.94
C-reactive protein	0.44	1.01	0.87–1.39
HbA1c	<0.001	0.096	0.03–0.29
Fasting glucose	<0.001	0.87	0.82–0.94
Serum creatinine	0.04	62.8	1.21–324
eGFR	0.001	1.09	1.03–1.14

**Table 3:** Data for diabetes mellitus patients with microvascular complications, diabetic patients without microvascular complications, and control group.

		T2DM with Microvascular Complications	T2DM without Microvascular Complications	Control Group	*p*
Sex	Women (n, %)	253 (59%)	163 (36.5%)	73 (29%)	<0.001
	Men (n, %)	173 (41%)	284 (63.5%)	181 (71%)	
		*Mean ± SD*			
Age (years)		59.3 ± 10.5	56.2 ± 10.4	47.2 ± 13.8	<0.001
Hb (g/dL)		13.1 ± 1.7	14.4 ± 2	13.9 ± 1.4	<0.001
		*Median (min–max)*			
PNI (%)		42.8 (30.1–62.5)	57.6 (43.1–73.8)	64.8 (49.4–76)	<0.001
Height (m)		1.6 (1.4–1.9)	1.66 (1.35–1.85)	1.67 (1.53–1.87)	0.099
Weight (kg)		80 (45–150)	90 (58–123)	72 (55–136)	<0.001
Waist circumference (cm)		105 (75–160)	107 (82–137)	95 (65–144)	<0.001
BMI (kg/m^2^)		30.5 (16.6–55.4)	31.3 (20.6–46.1)	27.6 (18.3–49.4)	<0.001
Systolic BP (mmHg)		120 (90–200)	125 (95–180)	120 (100–180)	0.11
Diastolic BP (mmHg)		75 (50–110)	80 (50–110)	80 (50–105)	0.44
Leukocyte count (k/mm^3^)		7 (4.2–14.4)	7.7 (4.2–13.7)	5.5 (4–14.1)	0.066
Htc (%)		39 (25–52)	42 (25–51)	42 (37–51)	0.037
Platelet count (k/mm^3^)		248 (154–441)	219 (158–423)	239 (151–374)	0.15
Albumin (g/dL)		3.2 (1.8–4.7)	4.4 (3.1–5.6)	4.4 (3.9–5.4)	<0.001
C-reactive protein (mg/L)		6.8 (0.1–25)	2.7 (0.1–17)	2.4 (0.1–12)	<0.001
HbA1c (%)		8.2 (5.9–17.2)	7.4 (6.1–15.9)	5.4 (4.8–6.4)	<0.001
Glucose (mg/dL)		170 (65–565)	127 (70–514)	93 (69–117)	<0.001
Urea (mg/dL)		32.1 (15–258)	32.1 (13–86)	26 (13–62)	0.057
Creatinine (mg/dL)		0.8 (0.5–3.93)	0.77 (0.39–1.87)	0.49 (0.4–1.3)	0.19
eGFR (%)		96 (14–110)	101 (45–111)	114 (58–118)	0.044
AST (U/L)		18 (6–97)	20 (8–53)	18 (9–35)	0.23
ALT (U/L)		19 (6–96)	26 (6–94)	19 (6–58)	0.21
Total cholesterol (mg/dL)		192 (50–318)	212 (50–378)	194 (114–290)	0.028
LDL-cholesterol (mg/dL)		112 (21–202)	129 (42–244)	112 (49–192)	0.019
HDL-cholesterol (mg/dL)		46 (13–92)	43 (17–77)	48 (21–85)	0.038
Triglyceride (mg/dL)		152 (47–1050)	171 (50–856)	124 (52–680)	<0.001

**Table 4:** Data for the diabetic patients, with and without diabetic nephropathy.

		T2DM with Diabetic Nephropathy	T2DM without Diabetic Nephropathy	*p*
Sex	Women (n, %)	164 (53%)	252 (44.5%)	0.01
	Men (n, %)	143 (47%)	314 (55.5%)	
		*Median (min–max)*		
Age (years)		57 (36–87)	60 (29–89)	0.42
Hb (g/dL)		13.1 (7.8–17.6)	14 (6.7–17.9)	<0.001
Htc (%)		39 (25–52)	41 (22–56)	<0.001
PNI (%)		43 (30–63)	56 (36–74)	<0.001
Height (m)		1.6 (1.4–1.9)	1.65 (1.35–1.85)	0.14
Weight (kg)		79 (45.5–150)	90 (58–123)	<0.001
Waist circumference (cm)		102 (75–160)	108 (82–148)	<0.001
BMI (kg/m^2^)		30 (17–49)	33 (21–55)	<0.001
Systolic blood pressure (mmHg)		120 (90–200)	130 (95–180)	0.07
Diastolic blood pressure (mmHg)		75 (50–110)	75 (50–110)	0.48
Leukocyte count (k/mm^3^)		7.2 (4.2–14.4)	7.2 (4.2–14)	0.28
Platelet count (k/mm^3^)		266 (154–441)	220 (151–374)	<0.001
Albumin (g/dL)		3.2 (1.8–4.7)	4.4 (2.9–5.6)	<0.001
C-reactive protein (mg/L)		6.5 (0.1–25)	3.4 (0.1–25)	<0.001
HbA1c (%)		8.9 (5.9–17.2)	7.4 (5.9–15.9)	<0.001
Glucose (mg/dL)		180 (65–565)	129 (66–514)	<0.001
Urea (mg/dL)		32 (15–258)	32 (13–222)	0.61
Creatinine (mg/dL)		0.8 (0.39–3.93)	0.78 (0.54–1.5)	0.99
eGFR (%)		95 (14–111)	102 (58–110)	<0.001
AST (U/L)		17 (6–97)	20 (6–58)	0.21
ALT (U/L)		19 (6–96)	23 (6–94)	0.56
Total cholesterol (mg/dL)		202 (52–318)	204 (50–378)	0.01
LDL-cholesterol (mg/dL)		112 (29–202)	126 (21–244)	0.003
HDL-cholesterol (mg/dL)		47 (13–92)	43 (14–80)	<0.001
Triglyceride (mg/dL)		152 (47–411)	166 (50–1050)	0.006

**Table 5:** Data of the diabetic patients, with and without diabetic retinopathy.

		T2DM with Diabetic Retinopathy	T2DM without Diabetic Retinopathy	*p*
Sex	Women (n, %)	60 (74%)	356 (45%)	<0.001
	Men (n, %)	21 (26%)	436 (55%)	
		*Median (min–max)*		
Age (years)		56 (46–89)	60 (29–86)	0.66
Hb (g/dL)		13.2 (6.7–16.9)	13.7 (8.5–17.9)	<0.001
Htc (%)		36 (22–49)	40 (25–56)	<0.001
PNI (%)		43.3 (36.3–59.8)	52.7 (30.1–73.8)	<0.001
Height (m)		1.61 (1.44–1.76)	1.65 (1.35–1.9)	0. 001
Weight (kg)		80 (55–120)	86 (46–150)	0.65
Waist circumference (cm)		107 (82–148)	105 (75–160)	0.15
BMI (kg/m^2^)		32.3 (22.2–55.4)	31.1 (16.6–49)	0.19
Systolic blood pressure (mmHg)		130 (100–170)	120 (90–200)	0.09
Diastolic blood pressure (mmHg)		80 (60–110)	75 (50–110)	0.2
Leukocyte count (k/mm^3^)		7.1 (4.6–14)	7.2 (4.2–14.4)	0.19
Platelet count (k/mm^3^)		225 (151–441)	235 (154–374)	0.25
Albumin (g/dL)		3.2 (2.9–4.7)	4.1 (1.8–5.6)	<0.001
C-reactive protein (mg/L)		8.1 (0.1–25)	3.6 (0.1–17)	<0.001
HbA1c (%)		9.3 (6.1–14.8)	7.4 (5.9–17.2)	<0.001
Glucose (mg/dL)		196 (66–439)	136 (65–565)	<0.001
Urea (mg/dL)		39 (20–222)	32 (13–258)	0.07
Creatinine (mg/dL)		0.79 (0.5–3.4)	0.8 (0.39–3.93)	0.7
eGFR (%)		100 (14–110)	102 (15–111)	0.054
AST (U/L)		17 (6–45)	18 (7–97)	0.56
ALT (U/L)		18 (6–42)	23 (12–94)	0.48
Total cholesterol (mg/dL)		196 (50–318)	205 (50–378)	0.34
LDL-cholesterol (mg/dL)		125 (21–197)	124 (42–244)	0.49
HDL-cholesterol (mg/dL)		44 (14–80)	44 (13–92)	0.58
Triglyceride (mg/dL)		143 (50–358)	157 (47–1050)	0.68

**Table 6:** Data of the diabetic patients, with and without diabetic neuropathy.

		T2DM with Diabetic Neuropathy	T2DM without Diabetic Neuropathy	*p*
Sex	Women (n, %)	184 (64%)	232 (40%)	<0.001
	Men (n, %)	104 (36%)	353 (60%)	
		*Median (min–max)*		
Age (years)		58.5 (41–87)	60 (29–89)	0.02
Hb (g/dL)		13.2 (7.8–16.9)	14 (6.7–17.9)	<0.001
Htc (%)		39 (25–49)	41 (22–56)	<0.001
PNI (%)		43.3 (36.3–59.8)	52.7 (30.1–73.8)	<0.001
Height (m)		1.6 (1.44–1.82)	1.66 (1.35–1.9)	<0.001
Weight (kg)		81 (48–120)	90 (46–150)	<0.001
Waist circumference (cm)		105 (75–132)	106 (78–160)	0.053
BMI (kg/m^2^)		30.5 (16.6–46.3)	31.2 (18.9–55.4)	0.31
Systolic blood pressure (mmHg)		120 (90–180)	125 (90–200)	0.16
Diastolic blood pressure (mmHg)		70 (50–110)	80 (50–110)	0.09
Leukocyte count (k/mm^3^)		6.9 (4.2–14)	7.5 (4.2–14.4)	0.11
Platelet count (k/mm^3^)		250 (155–441)	222 (151–374)	0.08
Albumin (g/dL)		3.2 (1.8–4.6)	4.4 (2.9–5.6)	<0.001
C-reactive protein (mg/L)		9.5 (0.1–25)	2.1 (0.1–21)	<0.001
HbA1c (%)		8.3 (5.9–16.5)	7.4 (6.1–17.2)	<0.001
Glucose (mg/dL)		180 (66–565)	128 (65–514)	<0.001
Urea (mg/dL)		32 (17–222)	32 (13–258)	0.16
Creatinine (mg/dL)		0.82 (0.6–3.4)	0.79 (0.39–3.93)	0.97
eGFR (%)		99.8 (14–105)	105 (15–111)	0.001
AST (U/L)		19 (6–69)	19 (8–97)	0.14
ALT (U/L)		20 (6–94)	23 (14–74)	0.11
Total cholesterol (mg/dL)		187 (52–318)	206 (50–378)	<0.001
LDL-cholesterol (mg/dL)		112 (21–200)	129 (29–244)	<0.001
HDL-cholesterol (mg/dL)		46 (13–87)	43 (17–92)	<0.001
Triglyceride (mg/dL)		153 (47–1050)	160 (50–856)	0.74

## Data Availability

Data related to this work are available from the corresponding author on reasonable request.
